# Elevated risk of chronic obstructive pulmonary disease in systemic lupus erythematosus: a population-based study

**DOI:** 10.1186/ar4650

**Published:** 2014-09-18

**Authors:** Antonio Avina-Zubieta, Mohsen Sadatsafavi, Eric C Sayre, John Esdaile

**Affiliations:** 1 University of British Columbia, Vancouver, BC, Canada; 2 Arthritis Research Centre of Canada, Richmond, BC, Canada; 3 Centre for Clinical Epidemiology and Evaluation, University of British Columbia, Vancouver, BC, Canada

## Background

Chronic obstructive pulmonary disease (COPD) has been recently recognized as an inflammatory disease. A recent Swedish hospital-based study found an increased risk of COPD in patients with a number of autoimmune conditions including systemic lupus erythematosus (SLE). We wonder whether the risk is also present in SLE patients from the general population. The objective was to assess the future risk of newly recorded COPD cases among incident SLE cases compared with controls from the general population using physician billing and hospitalization data that cover the entire province of British Columbia (BC), Canada.

## Methods

Our data include all health professionals and hospital visits covered by the comprehensive provincial medical services plan (1990 to 2010) and all dispensed medication (1996 to 2010), for all BC residents. We conducted a retrospective matched cohort (1996 to 2010) study among patients satisfying at least one of the following validated criteria: one diagnostic code for SLE (ICD-9-CM = 710.0) on at least two visits within a 2-year period by a nonrheumatologist physician; one ICD-9 code by a rheumatologist or from hospitalization; and absence of a prior SLE diagnosis between 1990 and 1995. Ten controls matched by birth year, sex and calendar year of exposure were randomly selected from the general population for each case. Outcome: we used a validated criteria to define COPD (first ICD-9-CM: 491, 492, 496, 493.2, or ICD-10-CM J43 or J44) from hospitals or death certificates. We estimated relative risks (RRs) by comparing SLE cases with age-matched, sex-matched and entry-time-matched comparison cohorts, adjusting for confounders. Sensitivity analyses were conducted to assess for unmeasured confounders.

## Results

Among 4,486 individuals with incident SLE, 96 developed COPD (incidence rate = 4.96 per 1,000 person-years) (Table 1). The age-matched, sex-matched and entry-time matched RRs were significantly increased in the SLE cohort when compared with controls (RR 2.31, 95% CI 1.83 to 2.89). After adjusting for covariates the results remained statistically significant. The risk of developing COPD was highest within the first year following the diagnosis of SLE, decreasing over time and remaining significant up to 4 years after diagnosis. Our results remained statistically significant after adjusting for the potential impact of an unmeasured confounder (adjusted RRs ranging between 1.58 and 1.98 in all sensitivity analyses).

**Table 1:** Risk of incident COPD according to SLE status

	SLE (*n *= 4,486)	Non-SLE (*n *= 47,190)
COPD cases, *n*	96	419
Incidence rate/1,000 person-years	5.0	2.1
Age-matched, sex-matched, and entry time-matched RRs (95% CI)	2.3 (1.8 to 2.9)	1.0
<1 year of disease duration	6.1 (4.0 to 9.2)	1.0
1to 4.9 years of disease duration	1.7 (1.2 to 2.5)	1.0
5+ years of disease duration	1.5 (0.9 to 2.3)	1.0
Multivariable RR (95% CI)	2.0 (1.5 to 2.6)	1.0

## Conclusions

This is the first general population-based study indicating a twofold increased risk of COPD in patients with SLE. The risk of developing COPD was highest within the first year, declining thereafter, suggesting the potential pathogenic role of inflammation in the development of COPD.

